# Synthesis, crystal structure and Hirshfeld surface analysis of a copper(II) complex involving 3-methyl­benzoate and 2,2′-bi­pyridine ligands

**DOI:** 10.1107/S2056989023006904

**Published:** 2023-08-15

**Authors:** Adnan Qadir

**Affiliations:** a Department of Chemistry, College of Science, Salahaddin University, Erbil 44001, Iraq; Universidad de Los Andes Mérida, Venezuela

**Keywords:** crystal structure, coordination compound, Hirshfeld surface analysis, 3-methyl­benzoate

## Abstract

3-Methyl­benzoic acid (3-mbH) and 2,2′-bi­pyridine (bipy) reacted with a cop­per(II) salt forming a new mixed ligand complex, aqua­(2,2′-bi­pyridine-κ^2^ *N*,*N*′)bis­(3-methyl­benzoato)-κ^2^ *O*,*O*′;κ*O*-copper(II) 0.68-hydrate, [Cu(C_8_H_7_O_2_)_2_(C_10_H_8_N_2_)(H_2_O)]·0.68H_2_O or [Cu(3-mb)_2_(bipy)(H_2_O)]·0.68H_2_O. The coord­ination environment of Cu^II^ is a distorted octa­hedron. The metal atom is attached to two 3-mb moieties, which bind in monodentate and bidentate fashions. One of the 3-mb units is disordered. The coordination environment is completed by one bipy ligand and a water mol­ecule. A second water mol­ecule is outside the coordination sphere of the Cu^II^ atom and its occupancy refined to 0.68. The structure consists of chains along the *b-*axis direction formed by complex units joined *via* hydrogen bonds between the coordinated water mol­ecule and an O atom of a coordinated 3-mb unit. Hirshfeld surface analysis indicates that the most abundant contacts are H⋯H (56.8%), H⋯C/C⋯H (21.7%) and H⋯O/O⋯H (13.7%).

## Abstract 2

A new copper(II) complex with 3-methyl­benzoate and 2,2′-bi­pyridine synthesized displays chains of hydrogen-bonded complex units along the *b* axis. Hirshfeld surface analysis indicates that H⋯H and H⋯C/C⋯H contacts are the most important inter­actions.

## 1. Chemical context

The coordination chemistry of mixed-ligand copper(II) complexes continues to be of inter­est. Copper is an important part of various metalloenzymes. It takes part in many metabolic processes such as iron metabolism, mitochondrial oxidative phospho­rylation and catecholamine production (Chen *et al.*, 2020 ▸; De Freitas *et al.*, 2003 ▸). Mixed-ligand copper(II) carboxyl­ates containing nitro­gen donor ligands have been reported to display a variety of pharmacological and superoxide dismutase activities. For example, the bis­(acetato)­bis­(imidazole)­copper(II) complex exhibits anti­tumor activity (Tamura *et al.*, 1987 ▸) and copper(II) salicylate with imidazoles have dismutase activities (Abuhijleh, 2010 ▸). Incorporating nitro­gen donor ligands in metal complexes has resulted in enhancement of the biological activity of these complexes (Patel *et al.*, 2012 ▸). It has been reported that the steric effect of a substituent on the phenyl group of carboxyl­ate ligands in metal complexes affects the coordination number of the metal, the geometry of the complex and the coordination mode of the ligand (Saini *et al.*, 2015 ▸). In our previous contribution, the Cu^II^ complex with 3-mb and *N*,*N*,*N*,*N*-tetra­methylethyl­enedi­amine (tmeda), [Cu(3-mb)_2_(tmeda)(H_2_O)_2_], was prepared and characterized by single-crystal X-ray diffraction. The complex was octa­hedral with 3-mb acting as monodentate (Kansız *et al.*, 2021 ▸). In view of the above information, a new Cu^II^ carboxyl­ate containing 2,2′-bi­pyridine was synthesized, characterized by X-ray crystallographic analysis and studied by Hirshfeld surface analysis. 
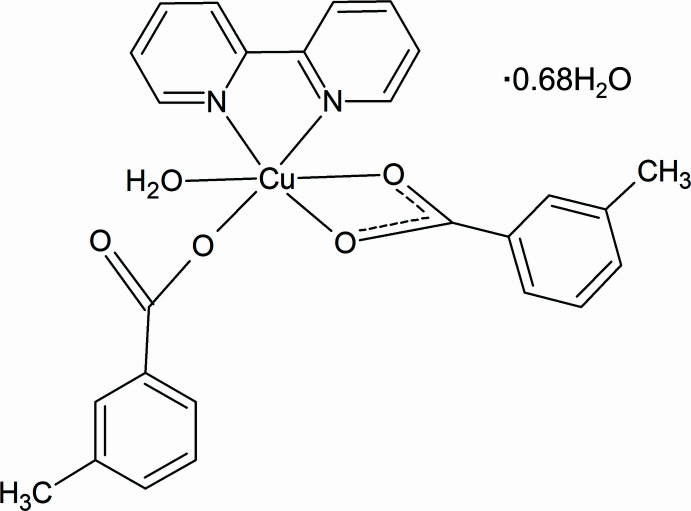


## 2. Structural commentary

**Figure 1:**
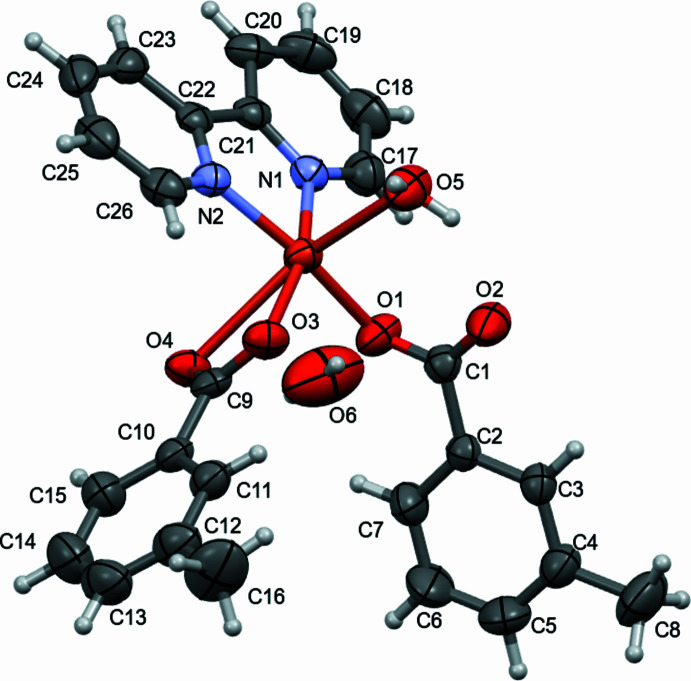
Mol­ecular structure of complex **1** with ellipsoids drawn at the 50% probability level. Only the major component of disorder is shown.

Complex **1** (Fig. 1 ▸) crystallizes in the monoclinic system in the *P*2_1_/*c* space group. The Cu^II^ atom has a distorted octa­hedral environment with the central copper atom coordinated by N_2_O_4_ donor sets. The Cu—N bond lengths range from 2.0071 (18) to 2.0131 (18) Å and the N1—Cu1—N2 angle is 80.58 (7)° (Table 1 ▸). The Cu1—O_carboxyl­ate_ distances are 1.842 (17)–2.2988 (18) Å. The Cu—O and Cu—N values are very close to those reported for copper(II) complexes involving benzoate (BZA) as a ligand, for example [Cu(BZA)_2_(bipy)(H_2_O)] [Cu—O = 1.9951 (12)–1.9633 (12) Å and Cu—N = 2.0064 (14)–2.0111 (13) Å; Devereux *et al.*, 2007 ▸]. This indicates that the presence of the methyl substituent has little or no effect on the Cu—O and Cu—N bond lengths. The 3-mb ligand defined by O3/O4/C9–C16 is disordered over two orientations related by an approximately 180° rotation.

## 3. Supra­molecular features

**Figure 2:**
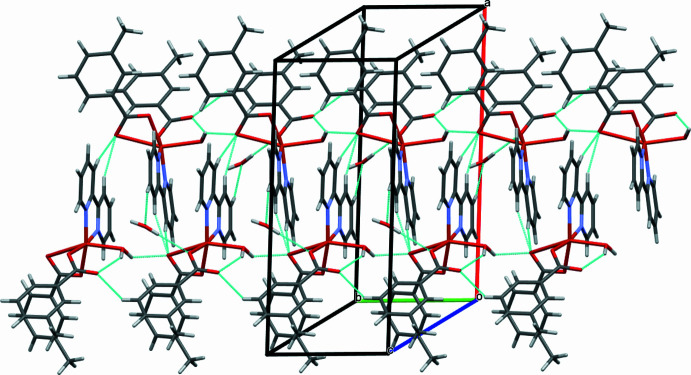
Partial view of the packing arrangement in compound **1** showing O—H⋯O inter­actions along the *b-*axis direction.

In the crystal, hydrogen bonding between H atoms of the coord­in­ated water mol­ecule and the O atoms of the coordinated 3-mb (O5—H5*B*⋯O4) leads to the formation of a linear chain in the *b*-axis direction (Fig. 2 ▸ and Table 1 ▸). The chains inter­digitate with other chains related by a screw-axis, connected *via* C—H⋯O inter­actions between O atoms of the 3-mb ligand and H atoms of the bipy ligand (Table 1 ▸), further consolidating the crystal. The occupancy of the solvent water (H6*A*—O6—H6*B*) refined to 0.68, which seems to be due to water escaping the crystal through the channels that run along the *b*-axis direction.

## 4. Database survey

A search of the Cambridge Structural Database (CSD, Version 5.42; Groom *et al.*, 2016 ▸) for compounds containing only Cu, O, N, C, and H resulted in 634 compounds containing bipy and 15 compounds containing 3-mb. In both lists, a dimeric compound containing bipy and 3-mb was identified (refcode PIGZAH; Li *et al.*, 2007 ▸). Other related compounds are AJEFEB (Wen, 2009 ▸), DUDYIN (He *et al.*, 2019 ▸), FERCOV (Wang *et al.*, 2005 ▸), GELXAX (Stephenson & Hardie, 2006 ▸), LEBOR (Tian *et al.*, 2011 ▸), QETNEJ (Chen *et al.*, 2006 ▸) and TOFZIZ (Gopalakrishnan *et al.*, 2014 ▸).

## 5. Hirshfeld surface analysis

**Figure 3:**
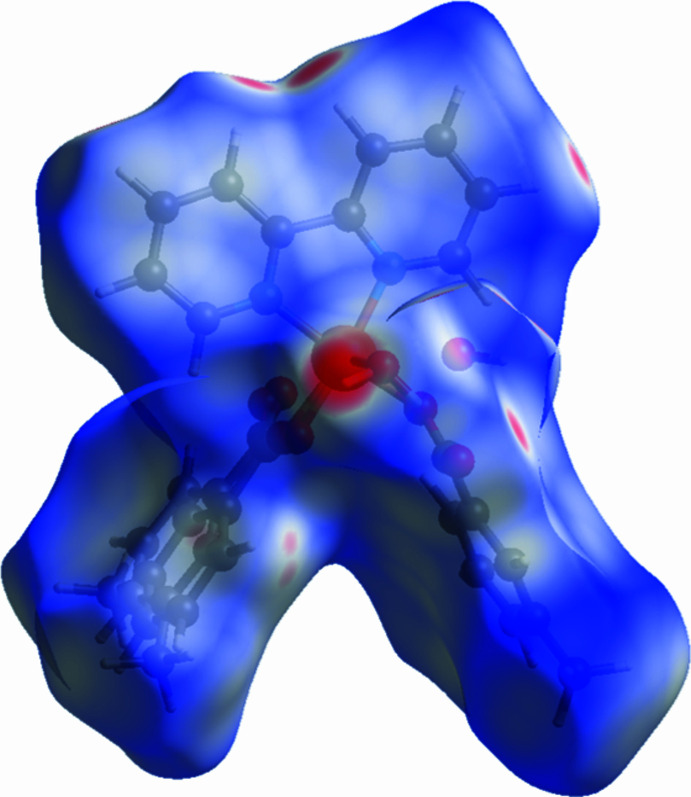
Hirshfeld surface map for the title complex.

**Figure 4:**
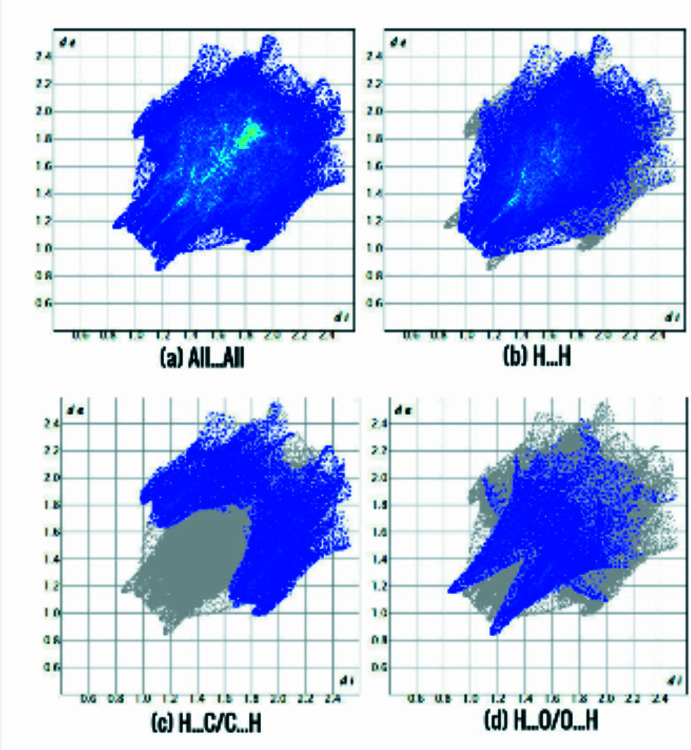
Fingerprint plot of the title compound showing all inter­actions and delineated into the most important inter­molecular contacts.

*CrystalExplorer* (Turner *et al.*, 2017 ▸) was used for Hirshfeld surface analysis and to generate the fingerprint plots. The purpose of using Hirshfeld surfaces, mapped onto *d*_norm_, is to provide additional insight into inter­molecular inter­actions. Close contacts shorter than van der Waals radii are shown as red spots on the surface. The closest contacts are responsible for directional supra­molecular inter­actions. The blue areas in the surface map represent weak contacts that are longer than the sum of the van der Waals radii. The Hirshfeld surface mapped onto *d*_norm_, is presented in Fig. 3 ▸. It displays several red spots due to O—H⋯O and C—H⋯O contacts. The intense spot near the coordinated water mol­ecule in the complex is assigned to the O5—H5⋯O hydrogen bond, as confirmed by the X-ray analysis (Table 1 ▸). Fingerprint plots for the contacts are shown in Fig. 4 ▸. The contributions of the H⋯H (Fig. 4 ▸ *b*), H⋯C/C⋯H (Fig. 4 ▸ *c*) and H⋯O/O⋯H (Fig. 4 ▸ *d*) contacts are 56.8, 21.7 and 13.7%, respectively.

## 6. Synthesis and crystallization

3-Methyl­benzoic acid (4 mmol, 0.54 g) and sodium hydroxide (4 mmol, 0.16 g) in water (20 ml) were added to a solution of Cu(NO_3_)_2_·3H_2_O (2 mmol, 0.48 g) in water (20 ml) under stirring. A solution of 2,2′-bi­pyridine (2 mmol, 0.3 g) in EtOH (25 ml) was added and the color changed from greenish blue to blue. The precipitate was filtered off, washed with water and dried. Blue single crystals of the title complex suitable for X-ray diffraction studies were obtained after evaporation of an ethanol solution after several days.

## 7. Refinement

Crystal data, data collection and structure refinement details are summarized in Table 2 ▸. One of the 3-methyl­benzoates (O3/O4/C9–C16) is disordered over two positions related by a 180° rotation. The occupancies of the two components refined to 0.664 (4):0.336 (4). The occupancy of the water mol­ecule H6*A*–O6–H6*B* refined to 0.680 (10). The coordinates of the ordered water atom were refined with *U*_iso_(H) = 1.5*U*_eq_(O). All other H atoms were positioned geometrically and refined as riding with *U*_iso_(H) = 1.2–1.5*U*_eq_(parent atom).

## Supplementary Material

Crystal structure: contains datablock(s) I. DOI: 10.1107/S2056989023006904/dj2063sup1.cif

Structure factors: contains datablock(s) I. DOI: 10.1107/S2056989023006904/dj2063Isup2.hkl

CCDC reference: 2117143

Additional supporting information:  crystallographic information; 3D view; checkCIF report

## Figures and Tables

**Table 1:** Hydrogen-bond geometry (Å, °)

*D*—H⋯*A*	*D*—H	H⋯*A*	*D*⋯*A*	*D*—H⋯*A*
O5—H5*A*⋯O2	0.85	1.93	2.643 (3)	140
O5—H5*B*⋯O4^a^ ^i^	0.85	2.09	2.694 (10)	128
C20—H20⋯O4^a^ ^ii^	0.93	2.40	3.324 (10)	171
C23—H23⋯O4^a^ ^ii^	0.93	2.51	3.405 (7)	163
C18—H18⋯O2^iii^	0.93	2.51	3.371 (4)	154
C24—H24⋯O6^ii^	0.93	2.36	3.200 (5)	151
C26—H26⋯O3^a^	0.93	2.48	2.984 (13)	115
C17—H17⋯O1	0.93	2.59	3.093 (3)	115
C7—H7⋯O6	0.93	2.72	3.405 (6)	131

Symmetry codes: (i) 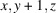 ; (ii) 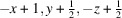 ; (iii) 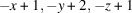 .

**Table 2:** Experimental details

Crystal data	
Chemical formula	[Cu(C_8_HH_7_O_2_)_2_(C_10_H_8_N_2_)(H_2_O)]·0.68H_2_O
*M*_r_	520.26
Crystal system, space group	Monoclinic, *P*2_1_/*c*
Temperature (K)	293
*a*, *b*, *c* (Å)	16.754 (3), 7.0021 (12), 22.103 (4)
β (°)	106.522 (6)
*V* (Å^3^)	2485.9 (8)
*Z*	4
Radiation type	Mo *K*α
μ (mm^−1^)	0.92
Crystal size (mm)	0.20 × 0.15 × 0.12

Data collection	
Diffractometer	Bruker APEXII CCD
No. of measured, independent and observed [*I* > 2σ(*I*)] reflections	63716, 6171, 4874
*R*_int_	0.035
(sin θ/λ)_max_ (Å^−1^)	0.670

Refinement	
*R*[*F* ^2^ > 2σ(*F* ^2^)], *wR*(*F* ^2^), *S*	0.042, 0.106, 1.09
No. of reflections	6171
No. of parameters	418
No. of restraints	347
H-atom treatment	H atoms treated by a mixture of independent and constrained refinement
Δρ_max_, Δρ_min_ (e Å^−3^)	0.30, −0.40

Computer programs: *APEX2* (Bruker, 2013 ▸), *SHELXT2018/2* (Sheldrick, 2015*a* ▸), *SHELXL2018/3* (Sheldrick, 2015*b* ▸) and *Mercury* (Macrae *et al*., 2020 ▸).

## supplementary crystallographic information

### Crystal data

**Table:**

[Cu(C_8_HH_7_O_2_)_2_(C_10_H_8_N_2_)(H_2_O)]·0.68H_2_O	*F*(000) = 1079
*M**_r_* = 520.26	*D*_x_ = 1.390 Mg m^−^^3^
Monoclinic, *P*2_1_/*c*	Mo *K*α radiation, λ = 0.71073 Å
*a* = 16.754 (3) Å	Cell parameters from 9233 reflections
*b* = 7.0021 (12) Å	θ = 2.5–26.6°
*c* = 22.103 (4) Å	µ = 0.92 mm^−^^1^
β = 106.522 (6)°	*T* = 293 K
*V* = 2485.9 (8) Å^3^	Block, blue
*Z* = 4	0.20 × 0.15 × 0.12 mm

### Data collection

**Table:**

Bruker APEXII CCD diffractometer	*R*_int_ = 0.035
φ and ω scans	θ_max_ = 28.5°, θ_min_ = 2.5°
63716 measured reflections	*h* = −22→22
6171 independent reflections	*k* = −9→9
4874 reflections with *I* > 2σ(*I*)	*l* = −29→29

### Refinement

**Table:**

Refinement on *F*^2^	Primary atom site location: dual
Least-squares matrix: full	Hydrogen site location: mixed
*R*[*F*^2^ > 2σ(*F*^2^)] = 0.042	H atoms treated by a mixture of independent and constrained refinement
*wR*(*F*^2^) = 0.106	*w* = 1/[σ^2^(*F*_o_^2^) + (0.0389*P*)^2^ + 1.6967*P*] where *P* = (*F*_o_^2^ + 2*F*_c_^2^)/3
*S* = 1.09	(Δ/σ)_max_ = 0.001
6171 reflections	Δρ_max_ = 0.30 e Å^−^^3^
418 parameters	Δρ_min_ = −0.40 e Å^−^^3^
347 restraints	

### Special details

**Table:**

Geometry. All esds (except the esd in the dihedral angle between two l.s. planes) are estimated using the full covariance matrix. The cell esds are taken into account individually in the estimation of esds in distances, angles and torsion angles; correlations between esds in cell parameters are only used when they are defined by crystal symmetry. An approximate (isotropic) treatment of cell esds is used for estimating esds involving l.s. planes.

### Fractional atomic coordinates and isotropic or equivalent isotropic displacement parameters (Å^2^)

**Table:**

	*x*	*y*	*z*	*U*_iso_*/*U*_eq_	Occ. (<1)
Cu1	0.34763 (2)	0.84495 (4)	0.31767 (2)	0.03879 (9)	
O1	0.32781 (10)	0.7602 (3)	0.39681 (8)	0.0519 (4)	
O2	0.26638 (14)	1.0161 (2)	0.42229 (9)	0.0664 (5)	
N1	0.47164 (11)	0.8619 (2)	0.35426 (8)	0.0391 (4)	
N2	0.38047 (11)	0.8817 (2)	0.23762 (8)	0.0377 (4)	
C1	0.27773 (13)	0.8419 (3)	0.42213 (10)	0.0392 (4)	
C2	0.23017 (13)	0.7151 (3)	0.45422 (9)	0.0372 (4)	
C3	0.18273 (14)	0.7973 (3)	0.48953 (10)	0.0422 (5)	
H3	0.182368	0.929463	0.493519	0.051*	
C4	0.13590 (17)	0.6878 (4)	0.51900 (12)	0.0563 (6)	
C5	0.1379 (2)	0.4918 (5)	0.51221 (15)	0.0735 (9)	
H5	0.107167	0.415128	0.531672	0.088*	
C6	0.1844 (2)	0.4077 (4)	0.47736 (17)	0.0801 (10)	
H6	0.184782	0.275525	0.473485	0.096*	
C7	0.23037 (18)	0.5186 (4)	0.44812 (13)	0.0571 (6)	
H7	0.261503	0.461430	0.424335	0.069*	
C8	0.0844 (2)	0.7820 (6)	0.55660 (18)	0.0969 (12)	
H8A	0.113429	0.775074	0.600751	0.145*	
H8B	0.031860	0.717351	0.548780	0.145*	
H8C	0.074998	0.913325	0.544171	0.145*	
O3	0.2315 (6)	0.7725 (11)	0.2675 (5)	0.0456 (16)	0.664 (4)
O4	0.2993 (4)	0.4976 (13)	0.2761 (4)	0.0443 (13)	0.664 (4)
C9	0.2338 (3)	0.5930 (10)	0.2581 (4)	0.0401 (12)	0.664 (4)
C10	0.1531 (2)	0.4981 (6)	0.2230 (2)	0.0427 (9)	0.664 (4)
C11	0.0787 (3)	0.5949 (7)	0.21325 (19)	0.0493 (9)	0.664 (4)
H11	0.079230	0.717697	0.229388	0.059*	0.664 (4)
C12	0.0028 (3)	0.5143 (9)	0.1799 (2)	0.0644 (12)	0.664 (4)
C13	0.0051 (3)	0.3318 (9)	0.1573 (3)	0.0746 (14)	0.664 (4)
H13	−0.044531	0.273815	0.135003	0.090*	0.664 (4)
C14	0.0781 (3)	0.2330 (8)	0.1666 (2)	0.0792 (13)	0.664 (4)
H14	0.077331	0.110027	0.150537	0.095*	0.664 (4)
C15	0.1529 (3)	0.3148 (7)	0.1996 (2)	0.0616 (11)	0.664 (4)
H15	0.202420	0.247440	0.206067	0.074*	0.664 (4)
C16	−0.0769 (3)	0.6241 (10)	0.1687 (3)	0.099 (2)	0.664 (4)
H16A	−0.100922	0.642619	0.124164	0.148*	0.664 (4)
H16B	−0.065780	0.745972	0.189242	0.148*	0.664 (4)
H16C	−0.115080	0.553947	0.185422	0.148*	0.664 (4)
O3'	0.2373 (12)	0.789 (2)	0.2796 (10)	0.040 (2)	0.336 (4)
O4'	0.2799 (8)	0.498 (3)	0.2621 (9)	0.047 (3)	0.336 (4)
C9'	0.2241 (6)	0.618 (2)	0.2594 (9)	0.042 (2)	0.336 (4)
C10'	0.1349 (5)	0.5677 (11)	0.2262 (4)	0.0430 (16)	0.336 (4)
C11'	0.1173 (5)	0.3862 (12)	0.2021 (4)	0.0552 (16)	0.336 (4)
H11'	0.160250	0.298071	0.207060	0.066*	0.336 (4)
C12'	0.0359 (6)	0.3330 (15)	0.1703 (6)	0.068 (2)	0.336 (4)
C13'	−0.0260 (6)	0.4669 (14)	0.1651 (5)	0.067 (2)	0.336 (4)
H13'	−0.080912	0.432829	0.145436	0.081*	0.336 (4)
C14'	−0.0089 (5)	0.6484 (14)	0.1879 (4)	0.0641 (19)	0.336 (4)
H14'	−0.051704	0.737327	0.182021	0.077*	0.336 (4)
C15'	0.0720 (4)	0.7003 (13)	0.2197 (4)	0.0503 (16)	0.336 (4)
H15'	0.083741	0.822434	0.236369	0.060*	0.336 (4)
C16'	0.0144 (7)	0.1356 (13)	0.1463 (5)	0.090 (3)	0.336 (4)
H16D	−0.002135	0.136810	0.101007	0.135*	0.336 (4)
H16E	−0.030539	0.088314	0.160979	0.135*	0.336 (4)
H16F	0.062117	0.054395	0.161428	0.135*	0.336 (4)
C17	0.51321 (16)	0.8587 (4)	0.41546 (12)	0.0524 (6)	
H17	0.483401	0.844910	0.444839	0.063*	
C18	0.59886 (18)	0.8753 (4)	0.43692 (14)	0.0647 (7)	
H18	0.626326	0.873196	0.479876	0.078*	
C19	0.64208 (17)	0.8947 (4)	0.39341 (15)	0.0650 (8)	
H19	0.699777	0.905457	0.406664	0.078*	
C20	0.60063 (14)	0.8983 (3)	0.33014 (13)	0.0507 (6)	
H20	0.629658	0.911990	0.300250	0.061*	
C21	0.51485 (13)	0.8811 (3)	0.31178 (11)	0.0369 (4)	
C22	0.46312 (13)	0.8851 (3)	0.24534 (10)	0.0370 (4)	
C23	0.49461 (16)	0.8923 (3)	0.19373 (12)	0.0492 (6)	
H23	0.551776	0.893392	0.199385	0.059*	
C24	0.4399 (2)	0.8979 (4)	0.13391 (13)	0.0593 (7)	
H24	0.459856	0.901430	0.098753	0.071*	
C25	0.35603 (19)	0.8982 (4)	0.12654 (12)	0.0591 (7)	
H25	0.318396	0.904644	0.086519	0.071*	
C26	0.32842 (16)	0.8888 (3)	0.17930 (11)	0.0501 (6)	
H26	0.271393	0.887348	0.174260	0.060*	
O5	0.33408 (13)	1.1632 (2)	0.33836 (11)	0.0676 (5)	
H5A	0.311059	1.174260	0.367935	0.101*	
H5B	0.299885	1.215072	0.306576	0.101*	
O6	0.4289 (3)	0.4219 (8)	0.4465 (3)	0.119 (2)	0.680 (10)
H6A	0.441 (6)	0.309 (10)	0.446 (4)	0.178*	0.680 (10)
H6B	0.435 (6)	0.490 (13)	0.478 (4)	0.178*	0.680 (10)

### Atomic displacement parameters (Å^2^)

**Table:**

	*U*^11^	*U*^22^	*U*^33^	*U*^12^	*U*^13^	*U*^23^
Cu1	0.03652 (14)	0.03638 (15)	0.04663 (16)	0.00129 (11)	0.01693 (11)	0.00307 (11)
O1	0.0532 (9)	0.0548 (10)	0.0559 (10)	0.0099 (8)	0.0286 (8)	0.0131 (8)
O2	0.1003 (15)	0.0395 (10)	0.0768 (13)	0.0003 (9)	0.0534 (12)	0.0043 (9)
N1	0.0404 (9)	0.0344 (9)	0.0418 (10)	−0.0003 (7)	0.0107 (8)	−0.0015 (7)
N2	0.0392 (9)	0.0334 (9)	0.0409 (9)	−0.0016 (7)	0.0118 (7)	0.0026 (7)
C1	0.0423 (11)	0.0412 (12)	0.0339 (10)	0.0002 (9)	0.0105 (8)	0.0004 (9)
C2	0.0405 (11)	0.0386 (11)	0.0321 (10)	−0.0017 (9)	0.0095 (8)	−0.0013 (8)
C3	0.0492 (12)	0.0418 (12)	0.0359 (11)	0.0028 (10)	0.0126 (9)	−0.0023 (9)
C4	0.0593 (15)	0.0674 (18)	0.0479 (14)	−0.0020 (13)	0.0244 (12)	−0.0018 (12)
C5	0.088 (2)	0.0661 (19)	0.080 (2)	−0.0222 (16)	0.0450 (17)	0.0024 (16)
C6	0.120 (3)	0.0390 (14)	0.097 (2)	−0.0164 (16)	0.056 (2)	−0.0061 (15)
C7	0.0755 (17)	0.0394 (13)	0.0652 (16)	−0.0015 (12)	0.0343 (14)	−0.0083 (12)
C8	0.104 (3)	0.112 (3)	0.102 (3)	0.003 (2)	0.073 (2)	−0.006 (2)
O3	0.0315 (18)	0.047 (2)	0.055 (4)	0.0020 (17)	0.007 (2)	0.005 (2)
O4	0.035 (3)	0.0400 (18)	0.057 (4)	0.002 (2)	0.011 (2)	0.006 (2)
C9	0.038 (2)	0.044 (2)	0.044 (2)	−0.006 (2)	0.0222 (19)	0.007 (2)
C10	0.0408 (19)	0.051 (2)	0.0411 (17)	−0.0051 (17)	0.0202 (15)	0.0005 (18)
C11	0.0437 (19)	0.063 (2)	0.0424 (18)	−0.005 (2)	0.0149 (15)	−0.0017 (19)
C12	0.049 (2)	0.092 (3)	0.049 (2)	−0.014 (2)	0.0094 (19)	−0.002 (2)
C13	0.066 (3)	0.096 (3)	0.058 (3)	−0.029 (3)	0.011 (2)	−0.010 (2)
C14	0.088 (3)	0.077 (3)	0.072 (3)	−0.023 (3)	0.023 (2)	−0.022 (2)
C15	0.064 (2)	0.059 (2)	0.065 (2)	−0.010 (2)	0.024 (2)	−0.0119 (19)
C16	0.041 (2)	0.159 (6)	0.085 (3)	0.001 (3)	0.000 (2)	−0.008 (4)
O3'	0.035 (4)	0.046 (4)	0.041 (5)	−0.001 (3)	0.015 (3)	−0.006 (3)
O4'	0.032 (5)	0.050 (4)	0.054 (7)	0.001 (4)	0.005 (4)	0.010 (4)
C9'	0.036 (3)	0.048 (4)	0.044 (3)	0.000 (3)	0.018 (3)	0.005 (3)
C10'	0.038 (3)	0.053 (4)	0.043 (3)	−0.007 (3)	0.020 (3)	0.000 (3)
C11'	0.051 (3)	0.065 (3)	0.053 (3)	−0.009 (3)	0.021 (3)	−0.003 (3)
C12'	0.058 (4)	0.084 (4)	0.060 (4)	−0.019 (4)	0.016 (3)	−0.005 (3)
C13'	0.054 (4)	0.089 (4)	0.055 (4)	−0.024 (4)	0.010 (3)	−0.002 (4)
C14'	0.046 (3)	0.087 (4)	0.058 (4)	−0.002 (4)	0.012 (3)	0.000 (4)
C15'	0.038 (3)	0.066 (4)	0.048 (3)	−0.002 (3)	0.014 (2)	−0.003 (3)
C16'	0.088 (6)	0.093 (7)	0.084 (6)	−0.028 (5)	0.017 (5)	−0.025 (5)
C17	0.0596 (15)	0.0513 (14)	0.0447 (13)	−0.0019 (12)	0.0123 (11)	−0.0024 (11)
C18	0.0619 (16)	0.0594 (17)	0.0576 (16)	−0.0058 (13)	−0.0075 (13)	−0.0029 (13)
C19	0.0416 (13)	0.0574 (16)	0.085 (2)	−0.0084 (12)	0.0003 (13)	0.0048 (15)
C20	0.0394 (12)	0.0397 (12)	0.0741 (17)	−0.0039 (9)	0.0179 (12)	0.0059 (11)
C21	0.0387 (10)	0.0227 (9)	0.0512 (12)	−0.0009 (8)	0.0160 (9)	0.0010 (8)
C22	0.0446 (11)	0.0198 (9)	0.0494 (12)	−0.0021 (8)	0.0177 (9)	0.0015 (8)
C23	0.0557 (14)	0.0368 (12)	0.0641 (16)	−0.0040 (10)	0.0312 (12)	−0.0008 (11)
C24	0.094 (2)	0.0431 (13)	0.0502 (15)	−0.0102 (13)	0.0362 (15)	−0.0023 (11)
C25	0.0802 (19)	0.0492 (14)	0.0423 (13)	−0.0130 (13)	0.0084 (13)	0.0026 (11)
C26	0.0501 (13)	0.0444 (13)	0.0507 (14)	−0.0057 (10)	0.0061 (11)	0.0054 (10)
O5	0.0899 (14)	0.0395 (9)	0.0894 (14)	0.0115 (9)	0.0516 (12)	0.0108 (9)
O6	0.103 (3)	0.113 (4)	0.157 (5)	0.023 (3)	0.064 (3)	0.043 (4)

### Geometric parameters (Å, º)

**Table:**

Cu1—O3'	1.842 (17)	C16—H16A	0.9600
Cu1—O1	1.9628 (16)	C16—H16B	0.9600
Cu1—N1	2.0072 (18)	C16—H16C	0.9600
Cu1—O3	2.011 (8)	O3'—C9'	1.275 (8)
Cu1—N2	2.0131 (18)	O4'—C9'	1.248 (8)
Cu1—O5	2.2988 (18)	C9'—C10'	1.509 (7)
O1—C1	1.269 (3)	C10'—C11'	1.377 (8)
O2—C1	1.235 (3)	C10'—C15'	1.381 (8)
N1—C17	1.334 (3)	C11'—C12'	1.396 (9)
N1—C21	1.346 (3)	C11'—H11'	0.9300
N2—C26	1.336 (3)	C12'—C13'	1.378 (9)
N2—C22	1.346 (3)	C12'—C16'	1.488 (10)
C1—C2	1.499 (3)	C13'—C14'	1.367 (10)
C2—C7	1.382 (3)	C13'—H13'	0.9300
C2—C3	1.387 (3)	C14'—C15'	1.387 (8)
C3—C4	1.385 (3)	C14'—H14'	0.9300
C3—H3	0.9300	C15'—H15'	0.9300
C4—C5	1.382 (4)	C16'—H16D	0.9600
C4—C8	1.510 (4)	C16'—H16E	0.9600
C5—C6	1.373 (4)	C16'—H16F	0.9600
C5—H5	0.9300	C17—C18	1.382 (4)
C6—C7	1.377 (4)	C17—H17	0.9300
C6—H6	0.9300	C18—C19	1.365 (4)
C7—H7	0.9300	C18—H18	0.9300
C8—H8A	0.9600	C19—C20	1.373 (4)
C8—H8B	0.9600	C19—H19	0.9300
C8—H8C	0.9600	C20—C21	1.383 (3)
O3—C9	1.277 (5)	C20—H20	0.9300
O4—C9	1.250 (5)	C21—C22	1.478 (3)
C9—C10	1.508 (5)	C22—C23	1.387 (3)
C10—C11	1.380 (5)	C23—C24	1.379 (4)
C10—C15	1.384 (5)	C23—H23	0.9300
C11—C12	1.395 (6)	C24—C25	1.367 (4)
C11—H11	0.9300	C24—H24	0.9300
C12—C13	1.377 (7)	C25—C26	1.373 (4)
C12—C16	1.499 (7)	C25—H25	0.9300
C13—C14	1.369 (7)	C26—H26	0.9300
C13—H13	0.9300	O5—H5A	0.8517
C14—C15	1.383 (6)	O5—H5B	0.8515
C14—H14	0.9300	O6—H6A	0.82 (7)
C15—H15	0.9300	O6—H6B	0.82 (7)
			
O3'—Cu1—O1	86.6 (7)	C10—C15—H15	120.4
O3'—Cu1—N1	170.4 (5)	C12—C16—H16A	109.5
O1—Cu1—N1	94.41 (7)	C12—C16—H16B	109.5
O1—Cu1—O3	91.9 (4)	H16A—C16—H16B	109.5
N1—Cu1—O3	164.9 (3)	C12—C16—H16C	109.5
O3'—Cu1—N2	96.6 (7)	H16A—C16—H16C	109.5
O1—Cu1—N2	168.42 (7)	H16B—C16—H16C	109.5
N1—Cu1—N2	80.58 (7)	C9'—O3'—Cu1	114.1 (12)
O3—Cu1—N2	90.7 (3)	O4'—C9'—O3'	124.5 (10)
O3'—Cu1—O5	98.8 (5)	O4'—C9'—C10'	119.3 (9)
O1—Cu1—O5	93.69 (7)	O3'—C9'—C10'	116.1 (9)
N1—Cu1—O5	90.69 (7)	C11'—C10'—C15'	120.5 (7)
O3—Cu1—O5	102.7 (2)	C11'—C10'—C9'	118.6 (7)
N2—Cu1—O5	96.79 (7)	C15'—C10'—C9'	120.9 (7)
C1—O1—Cu1	123.87 (15)	C10'—C11'—C12'	120.8 (8)
C17—N1—C21	118.7 (2)	C10'—C11'—H11'	119.6
C17—N1—Cu1	126.13 (17)	C12'—C11'—H11'	119.6
C21—N1—Cu1	115.18 (14)	C13'—C12'—C11'	117.7 (8)
C26—N2—C22	119.2 (2)	C13'—C12'—C16'	120.1 (8)
C26—N2—Cu1	125.89 (16)	C11'—C12'—C16'	122.0 (9)
C22—N2—Cu1	114.78 (14)	C14'—C13'—C12'	121.9 (8)
O2—C1—O1	124.6 (2)	C14'—C13'—H13'	119.1
O2—C1—C2	118.7 (2)	C12'—C13'—H13'	119.1
O1—C1—C2	116.66 (19)	C13'—C14'—C15'	120.1 (8)
C7—C2—C3	119.0 (2)	C13'—C14'—H14'	119.9
C7—C2—C1	121.8 (2)	C15'—C14'—H14'	119.9
C3—C2—C1	119.2 (2)	C10'—C15'—C14'	118.9 (8)
C4—C3—C2	121.8 (2)	C10'—C15'—H15'	120.5
C4—C3—H3	119.1	C14'—C15'—H15'	120.5
C2—C3—H3	119.1	C12'—C16'—H16D	109.5
C5—C4—C3	117.6 (2)	C12'—C16'—H16E	109.5
C5—C4—C8	121.9 (3)	H16D—C16'—H16E	109.5
C3—C4—C8	120.4 (3)	C12'—C16'—H16F	109.5
C6—C5—C4	121.5 (3)	H16D—C16'—H16F	109.5
C6—C5—H5	119.3	H16E—C16'—H16F	109.5
C4—C5—H5	119.3	N1—C17—C18	122.6 (3)
C5—C6—C7	120.2 (3)	N1—C17—H17	118.7
C5—C6—H6	119.9	C18—C17—H17	118.7
C7—C6—H6	119.9	C19—C18—C17	118.3 (3)
C6—C7—C2	119.9 (2)	C19—C18—H18	120.9
C6—C7—H7	120.1	C17—C18—H18	120.9
C2—C7—H7	120.1	C18—C19—C20	120.2 (2)
C4—C8—H8A	109.5	C18—C19—H19	119.9
C4—C8—H8B	109.5	C20—C19—H19	119.9
H8A—C8—H8B	109.5	C19—C20—C21	118.7 (2)
C4—C8—H8C	109.5	C19—C20—H20	120.7
H8A—C8—H8C	109.5	C21—C20—H20	120.7
H8B—C8—H8C	109.5	N1—C21—C20	121.6 (2)
C9—O3—Cu1	105.6 (6)	N1—C21—C22	114.54 (18)
O4—C9—O3	122.6 (5)	C20—C21—C22	123.8 (2)
O4—C9—C10	120.4 (5)	N2—C22—C23	121.0 (2)
O3—C9—C10	117.0 (5)	N2—C22—C21	114.65 (18)
C11—C10—C15	119.4 (4)	C23—C22—C21	124.4 (2)
C11—C10—C9	120.1 (4)	C24—C23—C22	119.0 (2)
C15—C10—C9	120.5 (4)	C24—C23—H23	120.5
C10—C11—C12	122.1 (5)	C22—C23—H23	120.5
C10—C11—H11	119.0	C25—C24—C23	119.7 (2)
C12—C11—H11	119.0	C25—C24—H24	120.2
C13—C12—C11	116.9 (5)	C23—C24—H24	120.2
C13—C12—C16	122.1 (5)	C24—C25—C26	118.8 (2)
C11—C12—C16	121.0 (5)	C24—C25—H25	120.6
C14—C13—C12	122.0 (5)	C26—C25—H25	120.6
C14—C13—H13	119.0	N2—C26—C25	122.4 (2)
C12—C13—H13	119.0	N2—C26—H26	118.8
C13—C14—C15	120.5 (5)	C25—C26—H26	118.8
C13—C14—H14	119.8	Cu1—O5—H5A	109.4
C15—C14—H14	119.8	Cu1—O5—H5B	109.3
C14—C15—C10	119.2 (5)	H5A—O5—H5B	104.4
C14—C15—H15	120.4	H6A—O6—H6B	126 (9)
			
Cu1—O1—C1—O2	−36.8 (3)	O4'—C9'—C10'—C15'	−176.9 (19)
Cu1—O1—C1—C2	143.68 (16)	O3'—C9'—C10'—C15'	−1 (2)
O2—C1—C2—C7	170.4 (2)	C15'—C10'—C11'—C12'	0.4 (13)
O1—C1—C2—C7	−10.0 (3)	C9'—C10'—C11'—C12'	−179.2 (10)
O2—C1—C2—C3	−7.7 (3)	C10'—C11'—C12'—C13'	−1.1 (17)
O1—C1—C2—C3	171.8 (2)	C10'—C11'—C12'—C16'	−177.2 (10)
C7—C2—C3—C4	0.3 (4)	C11'—C12'—C13'—C14'	2.3 (19)
C1—C2—C3—C4	178.4 (2)	C16'—C12'—C13'—C14'	178.5 (11)
C2—C3—C4—C5	0.1 (4)	C12'—C13'—C14'—C15'	−2.7 (17)
C2—C3—C4—C8	−179.4 (3)	C11'—C10'—C15'—C14'	−0.7 (13)
C3—C4—C5—C6	−0.2 (5)	C9'—C10'—C15'—C14'	178.9 (9)
C8—C4—C5—C6	179.3 (3)	C13'—C14'—C15'—C10'	1.9 (14)
C4—C5—C6—C7	0.0 (6)	C21—N1—C17—C18	0.3 (3)
C5—C6—C7—C2	0.4 (5)	Cu1—N1—C17—C18	−178.59 (19)
C3—C2—C7—C6	−0.5 (4)	N1—C17—C18—C19	−0.3 (4)
C1—C2—C7—C6	−178.6 (3)	C17—C18—C19—C20	0.3 (4)
Cu1—O3—C9—O4	−2.8 (14)	C18—C19—C20—C21	−0.3 (4)
Cu1—O3—C9—C10	177.9 (6)	C17—N1—C21—C20	−0.3 (3)
O4—C9—C10—C11	168.3 (9)	Cu1—N1—C21—C20	178.71 (16)
O3—C9—C10—C11	−12.4 (11)	C17—N1—C21—C22	−179.34 (19)
O4—C9—C10—C15	−12.5 (11)	Cu1—N1—C21—C22	−0.3 (2)
O3—C9—C10—C15	166.7 (9)	C19—C20—C21—N1	0.3 (3)
C15—C10—C11—C12	−0.5 (7)	C19—C20—C21—C22	179.3 (2)
C9—C10—C11—C12	178.6 (5)	C26—N2—C22—C23	−1.2 (3)
C10—C11—C12—C13	0.4 (8)	Cu1—N2—C22—C23	174.20 (16)
C10—C11—C12—C16	−178.6 (5)	C26—N2—C22—C21	178.66 (18)
C11—C12—C13—C14	−0.2 (9)	Cu1—N2—C22—C21	−5.9 (2)
C16—C12—C13—C14	178.7 (6)	N1—C21—C22—N2	4.1 (2)
C12—C13—C14—C15	0.2 (10)	C20—C21—C22—N2	−174.88 (19)
C13—C14—C15—C10	−0.2 (8)	N1—C21—C22—C23	−175.98 (19)
C11—C10—C15—C14	0.4 (7)	C20—C21—C22—C23	5.0 (3)
C9—C10—C15—C14	−178.7 (5)	N2—C22—C23—C24	0.6 (3)
O1—Cu1—O3'—C9'	−89.7 (18)	C21—C22—C23—C24	−179.2 (2)
N2—Cu1—O3'—C9'	79.1 (18)	C22—C23—C24—C25	0.7 (4)
O5—Cu1—O3'—C9'	177.1 (17)	C23—C24—C25—C26	−1.4 (4)
Cu1—O3'—C9'—O4'	−3 (3)	C22—N2—C26—C25	0.5 (3)
Cu1—O3'—C9'—C10'	−178.4 (11)	Cu1—N2—C26—C25	−174.38 (19)
O4'—C9'—C10'—C11'	3 (2)	C24—C25—C26—N2	0.8 (4)
O3'—C9'—C10'—C11'	178.4 (17)		

### Hydrogen-bond geometry (Å, º)

**Table:**

*D*—H···*A*	*D*—H	H···*A*	*D*···*A*	*D*—H···*A*
O5—H5*A*···O2	0.85	1.93	2.643 (3)	140
O5—H5*B*···O4^a^^i^	0.85	2.09	2.694 (10)	128
C20—H20···O4^a^^ii^	0.93	2.40	3.324 (10)	171
C23—H23···O4^a^^ii^	0.93	2.51	3.405 (7)	163
C18—H18···O2^iii^	0.93	2.51	3.371 (4)	154
C24—H24···O6^ii^	0.93	2.36	3.200 (5)	151
C26—H26···O3^a^	0.93	2.48	2.984 (13)	115
C17—H17···O1	0.93	2.59	3.093 (3)	115
C7—H7···O6	0.93	2.72	3.405 (6)	131

Symmetry codes: (i) *x*, *y*+1, *z*; (ii) −*x*+1, *y*+1/2, −*z*+1/2; (iii) −*x*+1, −*y*+2, −*z*+1.

## References

[bb1] Abuhijleh, A. L. (2010). *J. Mol. Struct.* **980**, 201–207.

[bb2] Bruker (2013). *APEX2.* Bruker AXS Inc., Madison, Wisconsin, USA.

[bb3] Chen, J., Jiang, Y., Shi, H., Peng, Y., Fan, X. & Li, C. (2020). *Eur. J. Physiol.* **472**, 1415–1429. 10.1007/s00424-020-02412-2 32506322

[bb4] Chen, P.-K., Che, Y.-X. & Zheng, J. M. (2006). *Chin. J. Struct. Chem.* (Jiegou Huaxue) **25**, 1427-1430.

[bb5] De Freitas, J., Wintz, H., Hyoun Kim, J., Poynton, H., Fox, T. & Vulpe, C. (2003). *BioMetals*, **16**, 185–197. 10.1023/a:1020771000746 12572678

[bb6] Devereux, M., O’Shea, D., O’Connor, M., Grehan, H., Connor, G., McCann, M., Rosair, G., Lyng, F., Kellett, A., Walsh, M., Egan, D. & Thati, B. (2007). *Polyhedron*, **26**, 4073–4084.

[bb7] Gopalakrishnan, M., Senthilkumar, K., Rao, P. R., Siva, R. & Palanisami, N. (2014). *Inorg. Chem. Commun.* **46**, 54–59.

[bb8] Groom, C. R., Bruno, I. J., Lightfoot, M. P. & Ward, S. C. (2016). *Acta Cryst.* B**72**, 171–179. 10.1107/S2052520616003954 PMC4822653 27048719

[bb9] He, X., Chen, F., Zhang, D., Li, Y., Yang, H.-L. & Zhang, X.-Q. (2019). *Z. Anorg. Allg. Chem.* **645**, 1341–1348.

[bb10] Kansız, S., Qadir, M. Q., Dege, N. & Faizi, S. H. (2021). *J. Mol. Struct.* **1230**, 129916–129916.

[bb21] Li, W., Li, C.-H., Yang, Y.-Q. & Kuang, Y.-F. (2007). *Wuji Huaxue Xuebao*, **23**, 1264.

[bb22] Macrae, C. F., Sovago, I., Cottrell, S. J., Galek, P. T. A., McCabe, P., Pidcock, E., Platings, M., Shields, G. P., Stevens, J. S., Towler, M. & Wood, P. A. (2020). *J. Appl. Cryst.* **53**, 226–235. 10.1107/S1600576719014092 PMC6998782 32047413

[bb11] Patel, M. N., Dosi, P. A. & Bhatt, B. S. (2012). *J. Coord. Chem.* **65**, 3833–3844.

[bb12] Saini, A., Sharma, R. P., Kumar, S., Venugopalan, P., Gubanov, A. I. & Smolentsev, A. I. (2015). *Polyhedron*, **100**, 155–163.

[bb13] Sheldrick, G. M. (2015*a*). *Acta Cryst.* A**71**, 3–8.

[bb14] Sheldrick, G. M. (2015*b*). *Acta Cryst.* C**71**, 3–8.

[bb15] Stephenson, M. D. & Hardie, M. J. (2006). *Dalton Trans. pp.* 3407–3417. 10.1039/b600357e 16832489

[bb16] Tamura, H., Imai, H., Kuwahara, J. & Sugiura, Y. (1987). *J. Am. Chem. Soc.* **109**, 6870–6871.

[bb17] Tian, D., Zhou, Y., Guan, L. & Zhang, H. (2011). *J. Coord. Chem.* **64**, 565–573.

[bb18] Turner, M. J., McKinnon, J. J., Wolff, S. K., Grimwood, D. J., Spackman, P. R., Jayatilaka, D. & Spackman, M. A. (2017). *Crystal­Explorer17*. The University of Western Australia.

[bb19] Wang, P., Moorefield, C. N., Panzer, M. & Newkome, G. R. (2005). *Chem. Commun.* pp. 465–467. 10.1039/b412055h 15654371

[bb20] Wen, G.-L. (2009). *Z. Krist. New Cryst. Struct.* **224**, 495–497.

